# Connections between redlining, food access, hypertension, diabetes, and obesity in Boston

**DOI:** 10.3389/fpubh.2025.1505462

**Published:** 2025-02-05

**Authors:** Farhad Mehrtash

**Affiliations:** School of Public Health, Harvard University, Boston, MA, United States

**Keywords:** food insecurity, hypertension, diabetes, health disparities, food policy, obesity, redlining

## Abstract

This paper explores how redlining has disproportionately impacted the Boston neighborhoods of Dorchester, Roxbury, and Mattapan. Initiated in the 1930s, the discriminatory practice of marking these neighborhoods as high-risk for lending has led to significant health inequities today. The paper focuses on how limited access to healthier foods in these areas contributes to a higher prevalence of chronic diseases such as hypertension and obesity, compared to wealthier neighborhoods. Additionally, the paper examines interventions aimed at reducing health disparities by improving affordability and access to nutritious foods. The findings underscore the need for comprehensive policies and interventions with community-based involvement to address food insecurity and health disparities that originated from redlining in Boston.

## Introduction to redlining

Redlining refers to the discriminatory practice of the Home Owners’ Loan Corporation (HOLC), which, during the Great Depression, drew literal red-ink lines around neighborhoods characterized by low-income, racial, or ethnic minority populations (1–3). The practice resulted in the systematic exclusion of these populations from access to financing for homes and businesses (1, 2). The health consequences of redlining remain prevalent today among marginalized communities. A systematic review and meta-analysis have shown that areas affected by redlining are associated with gunshot-related injuries, preterm birth, heat-related illnesses, and many chronic diseases such as diabetes and hypertension (1). This paper will explore how redlining in Boston has led to disparate access to nutritious foods and higher rates of diet-related health diseases in predominantly Black and Latinx neighborhoods, specifically Dorchester, Roxbury, and Mattapan.

## Racial and ethnic composition

In Dorchester, Roxbury, and Mattapan, the Black/African American population is significantly higher than Boston overall. In Mattapan, 74% of residents were Black/African American in 2015, compared to 23% in Boston, marking a significant increase from 20% in 1970 (4). Similarly, Roxbury’s Black/African American population was 53%, more than double Boston’s overall percentage (5). Dorchester had a slightly lower percentage at 44%, but this is still nearly twice Boston’s citywide proportion (6).

These neighborhoods also have significant Hispanic and foreign-born populations. In Mattapan, 16% of residents were Hispanic, up from 7% in 2000, and 35% were foreign-born, higher than Boston’s 27% (4). Roxbury and Dorchester have similar Hispanic populations, with 29 and 16%, respectively, and their foreign-born populations were 26% in Roxbury and 34% in Dorchester (5, 6).

## History of redlining and blockbusting in Boston

In 1938, the first redlining map of Boston was created by the HOLC who colored and rated neighborhoods into four grades below (3, 7–9).

- Green (A, or “Best”)—signifying the most desirable areas for lending.
- Blue (B, or “Still Desirable”)—representing still desirable for lending that were exclusively White and high-income neighborhoods.
- Yellow (C, or “Definitely Declining”)—indicating areas appraisers saw as becoming less desirable over time.
- Red (D, or “Hazardous”)—marking the origins of the term redlining by excluding residents from loan programs.

HOLC, for example, colored Roxbury red due to the “infiltration of negros” and “Negro heavily concentrated north of Ruggles St” (7, 9, 10). Sections of South End, North End, West End, South Boston, East Boston, Charlestown, Dorchester, Roxbury, Cambridge, Somerville, Revere, Chelsea, Everett, and Malden were also redlined (9). On the other hand, Brookline and the outer suburbs received higher HOLC grades (9).

Redlining was eventually outlawed under the Federal Housing Act following the assassination of Martin Luther King Jr. in 1968 and the subsequent riots in Roxbury; however, racial discrimination in mortgage lending persisted (7, 10, 11). For example, bankers in Boston created the Boston Banks Urban Renewal Group (B-BURG) mortgage program, which exploited practices like blockbusting (7). Blockbusting was used to instill fear in white homeowners, into panic selling their homes at lower prices due to the perceived “threat of a Black invasion” (12). This insinuated that Black residents moving into the neighborhood would increase crime rates and decrease property values (12).

BBURG lending areas were limited to specific sections of Dorchester, Roxbury, Mattapan, and all red areas (7, 10). Additionally, the Federal Reserve Bank in 1989 reported that banks approved loans in Roxbury, Mattapan, and sections of Dorchester at much lower rates than other neighborhoods in Boston (11).

This paper will focus on Dorchester, Roxbury, and Mattapan to illustrate the disparate access to healthy foods. Most Black and Latinx populations live in Dorchester, Roxbury, and Mattapan (7, 8, 10). The neighborhoods with the lowest opportunities for access to resources, economic prospects, and health outcomes are found in Roxbury and Dorchester in contrast to the highest opportunity areas, which include Beacon Hill, Back Bay, and sections of South End, North End, Cambridge, Somerville, Brookline, Milton, and the western suburbs (9). Studies suggest that limited access to healthy and affordable food directly contributes to higher rates of diet-related conditions, such as obesity, diabetes, and hypertension (13).

## Food insecurity

The 2021 report from the Mayor’s Office of Food Access Agenda (OFA) of the City of Boston revealed significant disparities in food insecurity (14). Roxbury, Dorchester, and Mattapan have higher rates of food insecurity at 23, 18.1, and 18% compared to the Boston average of 15%, a situation exacerbated by the pandemic (8, 14, 15). In contrast, areas like Beacon Hill, Back Bay, North End, West End, and Downtown maintain lower rates of food insecurity (14). The impact of historical redlining is evident in Dorchester, Roxbury, and Mattapan, where extensive areas of these neighborhoods are situated more than half a mile from a grocery store (14). Dorchester, Roxbury, and Mattapan also have smaller convenience stores, like corner stores, where access to fresh foods is limited (14). Conversely, neighborhoods like Back Bay, Downtown, Beacon Hill, North End, and West End boast a higher density and variety of food retailers, reflecting their superior availability of nutritional resources (14).

## Percent of adults reporting hunger

**Figure 1:**
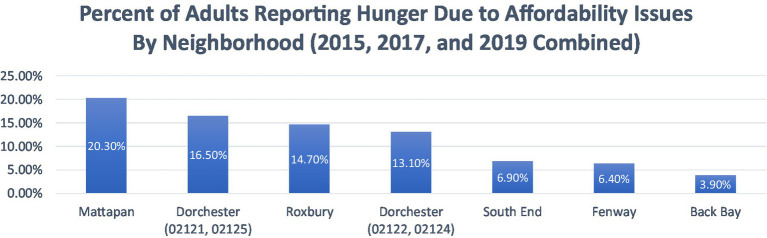
The percent of adults reporting hunger due to affordability issues by neighborhood (2015, 2017, and 2019 combined). Adapted from: Boston CHNA-CHIP Collaborative. Boston Community Health Collaborative—2022 Community Health Needs Assessment. Boston CHNA-CHIP (15).

Based on the Boston Community Health Needs Assessment—A Community Health Improvement Plan (CHNA-CHIP) Collaborative comparisons can be drawn about the percentage of adults who reported experiencing hunger due to affordability issues (15, 16). ‘Hunger’ was measured through self-reported experiences of not eating due to the inability to afford food as part of the Boston Behavioral Risk Factor Surveillance System survey (16). Respondents were asked if it was sometimes or often true in the past 12 months that they remained hungry because they could not afford food (16). This data was collected and combined for the years 2013, 2015, and 2017, and analyzed by neighborhood (16). For example, as shown in Figure 1, Mattapan, Dorchester, and Roxbury have a significant portion of the population reporting experiencing hunger due to affordability issues, representing some of the highest rates in the city (15). Conversely, residents living in Back Bay, Fenway, and South End had the lowest percentages (15).

## Hypertension prevalence and outcomes

**Figure 2:**
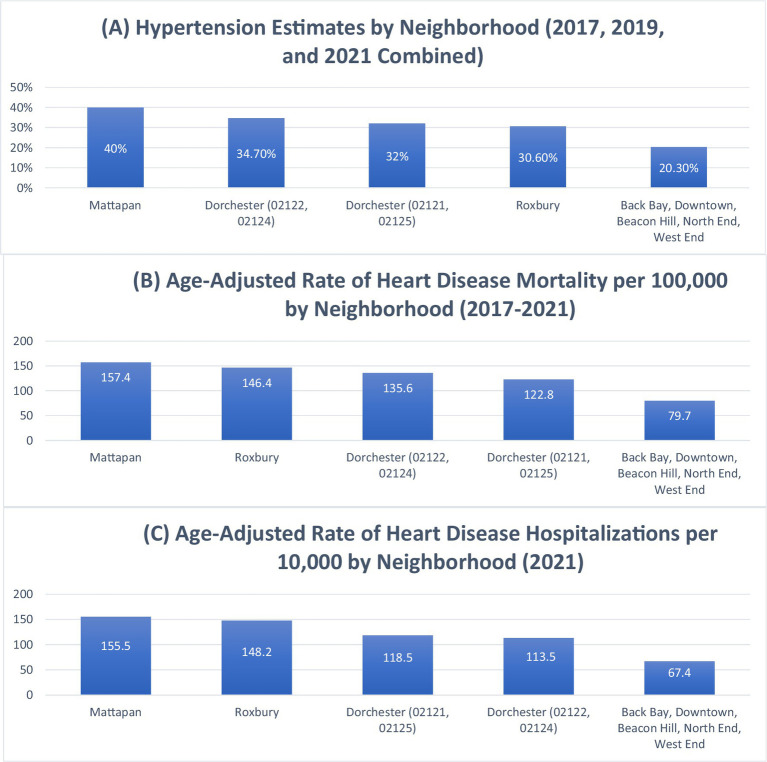
Hypertension prevalence, heart disease mortality, and heart disease hospitalization rates by neighborhood in Boston. This composite figure presents: **Section A**: Estimated prevalence of hypertension by neighborhood (2017, 2019, and 2021 combined). Data adapted from: Boston Public Health Commission (17). **Section B**: Age-adjusted rate of heart disease mortality per 100,000 by neighborhood (2017–2021). Data adapted from: Boston Public Health Commission (17). **Section C**: Age-adjusted rate of heart disease hospitalizations per 10,000 by neighborhood (2021). Data adapted from: Boston Public Health Commission (17).

To compare the prevalence of hypertension and the age-adjusted rates of heart disease hospitalization and mortality, the latest Heart Disease Report by the Boston Public Health Commission (BPHC) highlights disparities among neighborhoods, as shown in Figure 2, Sections A, B, and C (17). The combined areas of Back Bay, Downtown, Beacon Hill, North End, and West End exhibit some of the lowest prevalence of hypertension and age-adjusted rates of heart disease hospitalization and mortality in Boston (17). Conversely, Mattapan, Roxbury, and Dorchester report the city’s highest rates of these conditions (17). Black and Latinx residents disproportionately experience higher levels of such hypertension-related health outcomes compared to other races (17). The health impact of these food access disparities is evident in cardiovascular disease outcomes across neighborhoods, as illustrated in Figure 2.

## Diabetes prevalence and outcomes

**Figure 3:**
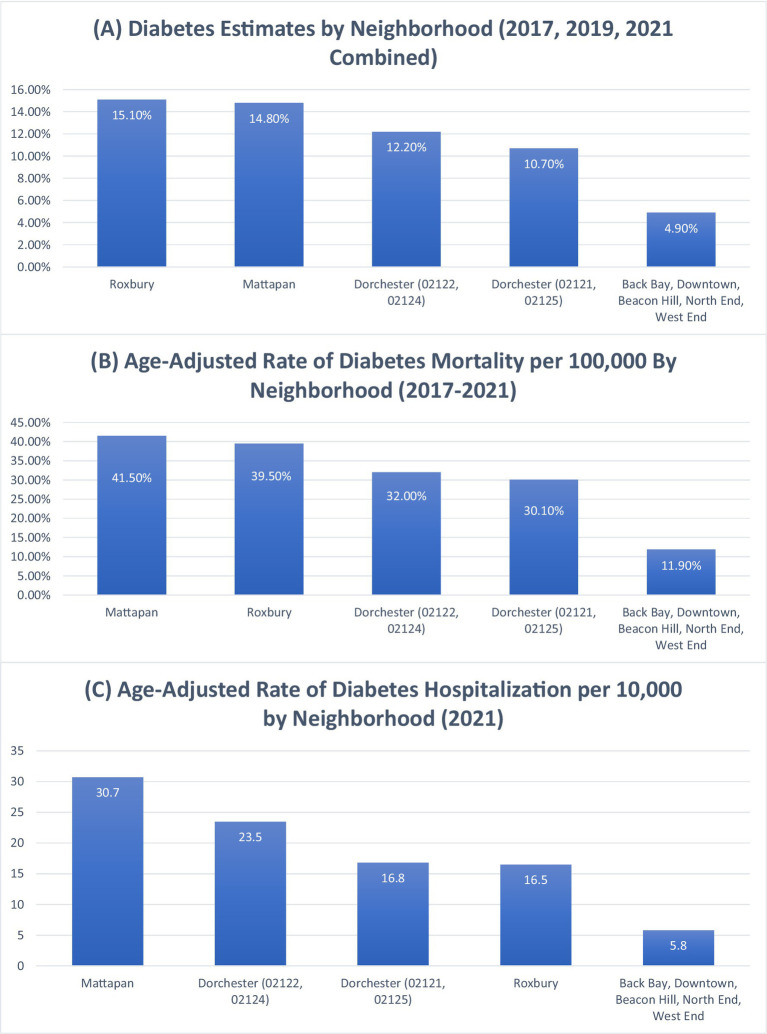
Diabetes prevalence, diabetes hospitalization, and diabetes mortality rates by neighborhood in Boston. This composite figure presents: **Section A**: Estimated prevalence of diabetes by neighborhood (2017, 2019, and 2021 combined). Data adapted from: Boston Public Health Commission (18). **Section B**: Age-adjusted rate of diabetes hospitalizations per 10,000 by neighborhood (2021). Data adapted from: Boston Public Health Commission (18). **Section C**: Age-adjusted rate of diabetes mortality per 100,000 by neighborhood (2017–2021). Data adapted from: Boston Public Health Commission (18).

The Boston Public Health Commission (BPHC) offers further insights into disparities in diabetes prevalence and the age-adjusted rates of diabetes hospitalization and mortality, as shown in Figure 3, Sections A, B, and C. Mirroring the trends observed with hypertension, the combined areas of Back Bay, Downtown, Beacon Hill, North End, and West End have some of the lowest diabetes prevalence and age-adjusted rates of diabetes hospitalization and mortality (18). Conversely, Mattapan, Roxbury, and Dorchester report the highest rates (18). Similar to hypertension patterns, Black and Latinx residents disproportionately experience higher levels of these diabetes-related health outcomes compared to other races (15, 16, 18). Similar patterns of health disparities exist in diabetes-related outcomes, shown in Figure 3 showing prevalence, hospitalizations, and mortality rates.

## Percent of adults reporting obesity or overweight

**Figure 4:**
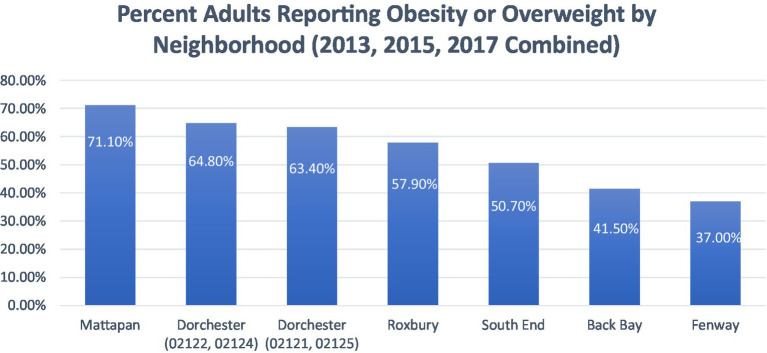
The percent of adults reporting obesity or overweight by neighborhood (2013, 2015, 2017 combined). Adapted from: Boston CHNA-CHIP Collaborative. Boston Community Health Collaborative—2022 Community Health Needs Assessment. Boston CHNA-CHIP (15).

The Boston CHNA-CHIP Collaborative also sheds light on disparities in obesity, as shown in Figure 4. Higher-income neighborhoods such as Back Bay, Fenway, and South End report the lowest percentages of adults classified as obese or overweight (15). In contrast, neighborhoods like Dorchester, Mattapan, and Roxbury report the highest percentages of adults classified as obese or overweight in the city (15). Echoing the trends seen with diabetes and hypertension, Black and Latinx residents disproportionately face higher levels of obesity and overweight compared to other races (15). The relationship between food access and health outcomes is further reflected in obesity rates, as shown in Figure 4.

## Discussion

This paper has methodological limitations that should be noted. The analysis including figures primarily relies on secondary data sources and descriptive statistics, without primary data collection. Furthermore, while the secondary data used comes from publicly available sources with established privacy protections, future studies should consider ethical implications including how findings may impact community representation and policy decisions. Future research would also greatly benefit from more rigorous methodological approaches (e.g., longitudinal cohort studies or mixed-methods research) incorporating both quantitative health data and especially qualitative community perspectives. These approaches including primary data collection and advanced statistical analyses (e.g., geospatial analysis), would help further validate the relationships between historical redlining and health outcomes.

While future research methodology improvements are needed, several immediate interventions could address current food access challenges. One potential initiative, considering the prevalence of smaller convenience stores scattered throughout Dorchester, Roxbury, and Mattapan, could involve increasing the availability of fresh foods within these existing stores. A similar strategy was implemented by Lawrence General Hospital, which invested $2.5 million in Lawrence, Massachusetts, for a project named “Healthy on the Block/Bodegas Saludables,” aimed at boosting the availability of healthier foods in bodegas (19–21). This initiative in Lawrence increased fruit and vegetable sales (19–21). A comparable intervention, “Healthy Bodegas,” in New York City also led to similar increases in purchasing healthier foods (22). Given that a higher intake of fruits and vegetables is associated with lower mortality rates, ensuring their wide availability in smaller convenience stores is crucial for people not to be limited to processed foods (23).

While the Supplemental Nutrition Assistance Program (SNAP) aids low-income communities, it is often insufficient. A U.S. Department of Agriculture (USDA) study found that 9 out of 10 participants encountered difficulty accessing healthy foods because of high costs (24, 25). This challenge is evident in Dorchester, Roxbury, and Mattapan, neighborhoods with the highest number of SNAP beneficiaries, indicating a greater need for assistance among individuals facing food insecurity (16, 24, 25).

A potential solution is to expand food prescription programs like Fresh Connect, which provides prepaid debit cards for purchasing healthier foods at grocery stores (26, 27). A unique aspect of Fresh Connect is that it provides a $100 monthly card exclusively for purchasing approved nutritious items such as fruits, vegetables, whole grains, and other healthy choices (28). Individuals experiencing food insecurity or living with chronic conditions such as hypertension, diabetes, and even children with autism, asthma, or obesity are eligible for Fresh Connect (28). Fresh Connect has received over $4 million in funding from MassHealth, USDA, Boston Medical Center, Mass General Brigham, and other philanthropic organizations (28). Currently, Fresh Connect is well-integrated in Dorchester and Roxbury, available at Stop & Shop and Daily Table stores and 16 farmers’ markets (28). Considering the significant impact of nutrition on health outcomes and the potential cost-effectiveness of food prescriptions as an alternative to pharmaceutical treatments for chronic conditions, it’s imperative to increase funding for such initiatives (23, 24, 29).

### Social determinants and barriers

While improving food access is crucial, the Boston COVID-19 Consultant Equity and Recovery Team [CERT] & Boston Health Inequities Taskforce Health Equity Now Plan [HITF] identify many other social determinants that impact health outcomes in redlined neighborhoods. Digital health barriers can impact older adults and low-income residents in Dorchester, Roxbury, and Mattapan, where limited digital literacy and connectivity challenges could restrict access to healthcare services like telemedicine (30). According to the PEW Research Center, only 56% of lower-income households have home broadband compared to 94% of higher-income households, limiting access to telemedicine, employment opportunities, and educational resources (30).

Cultural and linguistic barriers also affect healthcare access. With significant foreign-born populations (26% in Roxbury, 34% in Dorchester, 35% in Mattapan), many residents face challenges navigating English-centric healthcare systems (4–6). Those who speak or read a language other than English struggle to access services due to difficulties navigating the healthcare system including communication barriers with providers (30). Beyond communication challenges, these social determinants compound with poverty, resulting in limited healthcare access, increased stress and anxiety, and poor living conditions (30).

Communities of color disproportionately experience chronic diseases due to ‘allostatic load’—“perpetual high-stress levels that increase risk factors like blood pressure and affect diet choices” (30). This stress burden is amplified by systemic inequities in Boston as the Department of Health and Human Services, Boston Public Health Commission (BPHC) (30) indicate “an inherent power dynamic and disproportionate use of force exists in Boston’s neighborhoods of color, which undermines trust and perpetuates racial trauma that causes stress and increases allostatic load.” Further evidence from Risica et al. (31) showed how feelings of deprivation, anxiety, and race-related stress in Black women about securing adequate food, especially in unhealthy food environments trigger emotional eating and an increased risk of obesity. As a result, stress-induced emotional eating is often associated with the consumption of calorie-dense foods (31).

Additionally, Oshri et al. (32) found that racial discrimination activates the amygdala in Black youth, influencing anxiety and depression, which can increase stress-related eating behaviors (31, 32). Black male youth exposed to police-related deaths experience 46% higher cortisol levels, indicating how physiological stress can deter outdoor activity (33). The intersection of these factors creates an entrenched system of health disparities requiring comprehensive policy solutions beyond food access alone.

### Current limitations and solutions

A limitation of solely increasing healthier foods and providing funding for food purchasing is that it leaves several levels of the food system unaddressed in the fight against obesity, hypertension, and diabetes. For instance, obesity is multifaceted, and approaches that target individual, community, and system levels are more successful than single interventions (34).

Interventions should consider the systemic nature of chronic diseases like obesity and its comorbidities. To address the limitations of individual-level interventions, multi-level approaches can target systemic issues in food access and health outcomes. These interventions consider the complex interplay of individual, community, and policy factors that contribute to health disparities. By addressing multiple levels simultaneously, these approaches aim to create sustainable changes in food environments and health behaviors. The following examples demonstrate how multi-level interventions may effectively tackle systemic issues in ways that individual-level approaches limit.

An example of a multi-level approach intervention was conducted by researchers at Tufts University called “Shape Up Somerville (SUS)” (35). SUS successfully reduced the consumption of sugar-sweetened beverages, lowered body mass index in parents and children, and increased physical activity by targeting environmental, individual, and policy levels (35).

A similar intervention was conducted in Baltimore called “B’More Healthy Communities for Kids (BHCK)” by researchers at the Johns Hopkins Bloomberg School of Public Health (36). BHCK was a multilevel childhood obesity prevention program that helped decrease the intake of sweet snacks among low-income Black/African American youth (36). A combination of strategies lead to their success, including nutrition education sessions in recreation centers and in-store point-of-purchase promotions with repeated taste tests of healthier snacks (36).

The Stanford Five-City Project involved extensive collaboration with media, health organizations, schools, and other community stakeholders to deliver a 6-year multi-faceted health education program (37, 38). Using television, radio, print materials, classes, and events tailored to the community, the intervention significantly improved heart disease knowledge, attitudes, and risk behaviors, leading to reductions in smoking, cholesterol, and blood pressure (37, 38).

The Massachusetts Childhood Obesity Research Demonstration project guided by researchers at the Harvard School of Public Health and the Massachusetts General Hospital also showed important results (34). The study demonstrated how gathering input from numerous community stakeholders in two predominantly non-Hispanic white, low-income areas in Massachusetts through interviews helped implement an intervention focused on reducing obesity (34).

While many of these examples include some individual behavior modification, they demonstrate the need to address multiple levels of the food system collectively. Addressing the systemic health inequities in historically redlined neighborhoods also requires policy interventions that integrate healthcare, nutrition, and behavioral strategies into broader public health systems.

The proposed Medical Nutrition Therapy (MNT) Act of (39) (H.R. 6,407/S.3297) seeks to expand Medicare Part B coverage to include a range of conditions such as obesity, cardiovascular disease, celiac disease, and cancer (39). The MNT Act also aims to broaden eligibility for providers, including physician assistants, nurse practitioners, and clinical psychologists (39). By addressing gaps in coverage, the Act has the potential to empower healthcare providers to deliver MNT by directly addressing diet-related conditions prevalent in neighborhoods like Dorchester, Roxbury, and Mattapan. However, it remains unpassed and faces implementation challenges such as integration into Medicare Advantage plans and ensuring equitable access across diverse populations (39).

Another promising initiative is Food as Medicine (FIM) programs, which integrate nutrition-based interventions, such as medically tailored meals, groceries, and produce prescriptions, into healthcare systems (40). Recent evidence highlights the growing adoption of FIM programs, with 422 Medicare Advantage plans covering medically tailored meals and seven states (Arkansas, Delaware, Massachusetts, New Jersey, North Carolina, Oregon, Washington) integrating these interventions through Medicaid Section 1115 waivers (40). FIM programs have shown significant improvements in food security, diet quality, and clinical outcomes, including reductions in HbA1c (−0.8%) and BMI (0.6 kg/m^2^), with projected cost savings of $13.6 billion annually (40). Despite their potential, barriers such as limited screening processes, reimbursement challenges, and insufficient provider education may hinder the full adoption and scaling of FIM programs especially in underserved communities (40).

In addition to MNT and FIM initiatives, Intensive Behavioral Therapy (IBT) is another evidence-based approach to improving weight loss and glycemic control among individuals with diabetes and obesity (41). Medicare and the Affordable Care Act (ACA) have expanded IBT coverage, including telemedicine approval during the COVID-19 pandemic (41). However, utilization rates remain low, with less than 1% of eligible patients accessing IBT due to limited provider training, strict eligibility criteria, and time constraints (41). For example, Centers for Medicare and Medicaid Services (CMS) requirements set a BMI threshold of ≥30 for coverage (41). As a result, many additional barriers are created for individuals who are overweight and ethnic groups with lower obesity thresholds, forcing some patients to delay treatment until they gain more weight to qualify (41).

While implementation challenges remain, many of these initiatives underscore the importance of integrating nutrition and behavioral health strategies into broader public health frameworks to create sustainable solutions for marginalized communities.

## Conclusion

This brief research report has elucidated how historical redlining and blockbusting in Boston’s neighborhoods of Dorchester, Roxbury, and Mattapan have created persistent health inequities. Findings from this report show these neighborhoods face significantly higher rates of food insecurity (18–23% versus Boston’s 15% average), higher prevalence of chronic diseases, and disproportionate health burdens among Black and Latinx residents (14, 15, 42). Beyond food access, additional challenges include digital health disparities, language barriers among foreign-born populations (26–35%) compounded with socioeconomic complications that further exacerbate health outcomes including chronic stress (4–6, 30).

Future policy measures should address these multiple layers of disadvantage through preventative strategies such as expanding Medicare coverage under the proposed MNT Act, integrating FIM programs into healthcare systems, or increasing the utilization of IBT for weight management. These policies must be complemented by efforts to improve healthy food availability and affordability while bridging divides in healthcare access. Interventions should consider the interconnected nature of these challenges, addressing food access alongside the multiple social, economic, and structural barriers that affect healthcare access and outcomes. Ultimately, solutions must be developed through direct collaboration with community members and organizations in Dorchester, Roxbury, and Mattapan, ensuring interventions are culturally tailored and community-driven.

## Data Availability

The original contributions presented in the study are included in the article/supplementary material, further inquiries can be directed to the corresponding author.
